# Ultraviolet light scattering by a silicon Bethe hole

**DOI:** 10.1515/nanoph-2023-0557

**Published:** 2023-11-06

**Authors:** Dukhyung Lee, Youjin Lee, Dai-Sik Kim

**Affiliations:** Ulsan National Institute of Science and Technology, Ulsan 44919, Republic of Korea; Seoul National University, Seoul 08826, Republic of Korea

**Keywords:** scattering, Bethe hole, magnetic dipole, UV plasmonics, silicon

## Abstract

Bethe’s theory predicts that scattering by a small hole on a thin perfect electric conductor (PEC) is presented as radiation by an in-plane magnetic dipole of the incident magnetic field direction. Even in the near-infrared range where metals are no more PEC, the magnetic dipole radiation of Bethe holes has been demonstrated. However, such Bethe holes’ nature has not been addressed yet in the ultraviolet (UV) range where conductivity of metals becomes severely deteriorated. Meanwhile, UV plasmonics has been elevating its importance in spectroscopy and photochemistry, recognizing silicon (Si) as an alternative plasmonic metal featuring the interband transition in the UV range. In this work, we expanded the Bethe’s theory’s prediction to the UV range by investigating Si Bethe holes theoretically and experimentally in terms of the scattering pattern and polarization. Simulation results showed that the scattered field distribution resembles that of an in-plane magnetic dipole, and the dipole direction at oblique incidence is roughly given as the incident magnetic field direction with a deviation angle which can be predicted from the Fresnel equations. Simulation with various diameters showed that the magnetic dipole nature maintains with a diameter less than the quarter-wavelength and multipoles becomes noticeable for diameters larger than the half-wavelength. We performed scattering polarization measurement at 69-degree incidence, which confirms the theoretical analysis. The features of Si Bethe holes demonstrated here will be useful for designing UV plasmonic metasurfaces.

## 1 Introduction

Scattering by a small circular hole on a conducting film has been a classical problem treated in textbooks in the fields of optics and photonics, yet its usage is increasing, especially in the field of nanophotonics with ability to support localized surface plasmons. Due to the enhancement of electric field inside and light–matter interaction [1, 2], small circular holes have been exploited in various areas such as perfect absorber [3], single photon emission [4], single molecule fluorescence [5, 6], and surface-enhanced Raman spectroscopy [7]. Theory on scattering by a small circular hole given by Bethe in 1944 tells that scattering by a small hole is represented as in-plane magnetic and out-of-plane electric dipole radiations [8]. Although Bethe’s theory assumes a hole on a PEC screen, scattering polarization analysis on a near-infrared frequency revealed that a small hole can be regarded a magnetic dipole induced by the in-plane component of the incident magnetic field even at the plasmonic regime [9–11], which can be attributed to the fact that the permittivity of few tens is still large enough to approximate the surface current as **K** ≅ **n** × 2**H**_in_ where **H**_in_ is the incident magnetic field and **n** is the unit vector normal to the film [9, 12, 13]. This magnetic dipole response as a Bethe hole implies that we can analyse optical magnetic field by measuring scattering from a nano-sized hole, enabling magnetic field mapping with nanometer spatial resolution [10, 11].

UV plasmonics is an emerging frontier of photonics which finds interesting applications including spectroscopy [14, 15], photocatalysis [16], wavefront control [17, 18], and UV lasers [19, 20]. The fact that noble metals widely used for plasmonics in the visible and infrared ranges suffers from the low refractive indexes in the UV range made researchers in this field to search for alternative plasmonic materials. Along with several alternative metals such as aluminum [14, 21], gallium [22], and rhodium [15], Si have been suggested as a UV plasmonic material due to the considerable permittivity arising from the interband transition [17, 23]. An important advantage of Si is that Si nanostructures can be readily fabricated by the industrial fabrication techniques. In the recent years, some studies have demonstrated UV plasmonic systems using basic Si structures such as nanodisks and nanoholes [23] as well as sophisticated Si metasurfaces and metamaterials [17, 24], yet much remains to be proved in UV plasmonics using Si nanostructures. In this work, we studied UV scattering from Si nanoholes and demonstrated the features as a Bethe hole. The scattering pattern and polarization with normal or oblique UV incidence was theoretically investigated to reveal the magnetic dipole response and experimental measurement on scattering polarization was consistent with the simulation. Our results indicated that the same argument for metallic nanoholes can be applied to Si nanoholes, making it easier to expand the horizon of plasmonics to UV range using Si nanostructures.

## 2 Theoretical analysis

**Figure 1:**
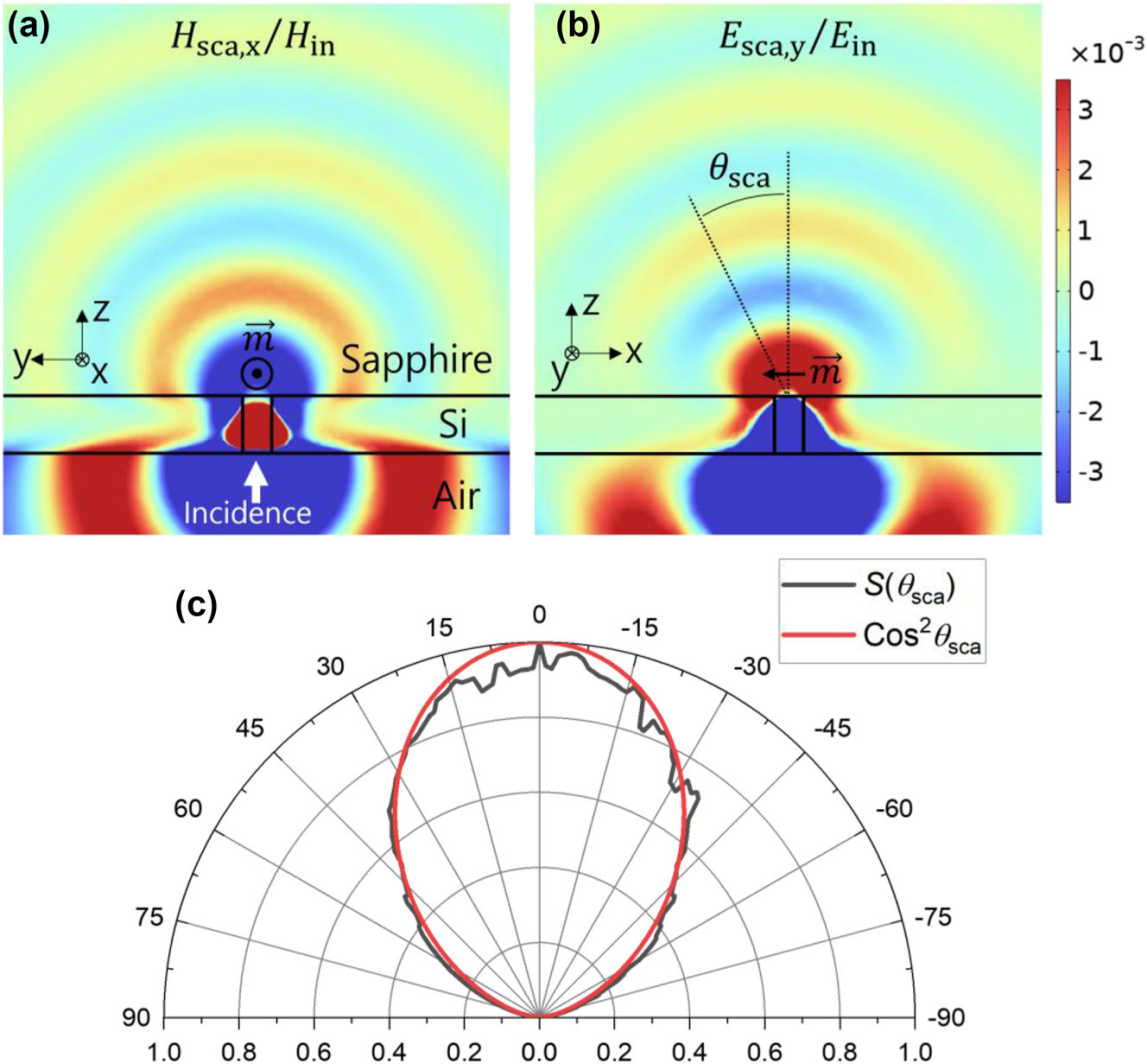
Simulation results on scattered fields for normal incidence of 310 nm UV plane wave. (a) Scattered magnetic field distribution in the *yz* plane. *y*-Polarized UV was incident from the air side. The Si thickness and hole diameter were 80 and 40 nm, respectively. (b) Scattered electric field distribution in the *xz* plane. (c) Polar plot of the normalized scattering intensity $S\left(\theta_{\text{sca}}\right)$ in the xz plane (black). Cosine squared (red) was also plotted for comparison.

The main goal of this work is to confirm that a Si small hole acts as an in-plane magnetic dipole induced by the in-plane component of the incident magnetic field in the UV range. One critical feature of magnetic dipole-like scattering is the radiation pattern given by cosine squared with zero at the magnetic dipole direction. Therefore, we started from examination of the scattering pattern. Figure 1(a) and (b) shows the simulation results for the scattered magnetic and electric field distributions respectively when *y*-polarized 310 nm UV plane wave is normally incident on a Si aperture of 40 nm diameter and 80 nm thickness on a sapphire substrate from the air side. Simulation details are given in the methods section. Compared to the magnetic field in the *yz* plane (Figure 1(a)), the electric field in the *xz* plane (Figure 1(b)) shows evident decrease with increasing scattering angle, implying magnetic dipole-like scattering induced by incident magnetic field of *x*-direction. To confirm this more clearly, we plotted scattering intensity *S* in the *xz* plane as a function of scattering angle *θ*_sca_ in Figure 1(c). The cosine squared function (red line, Figure 1(c)) expected for a dipole radiation agrees well with the simulated scattering pattern (black line, Figure 1(c)), except for the jiggling simulation artifact. Although an electric dipole directed along the *x*-axis can produce the same scattering intensity distribution, the fact that the incidence is *y*-polarized excludes that possibility. Investigation on scattered field components confirms again that the scattering is magnetic dipole-like (see Supplementary Materials S1).

**Figure 2:**
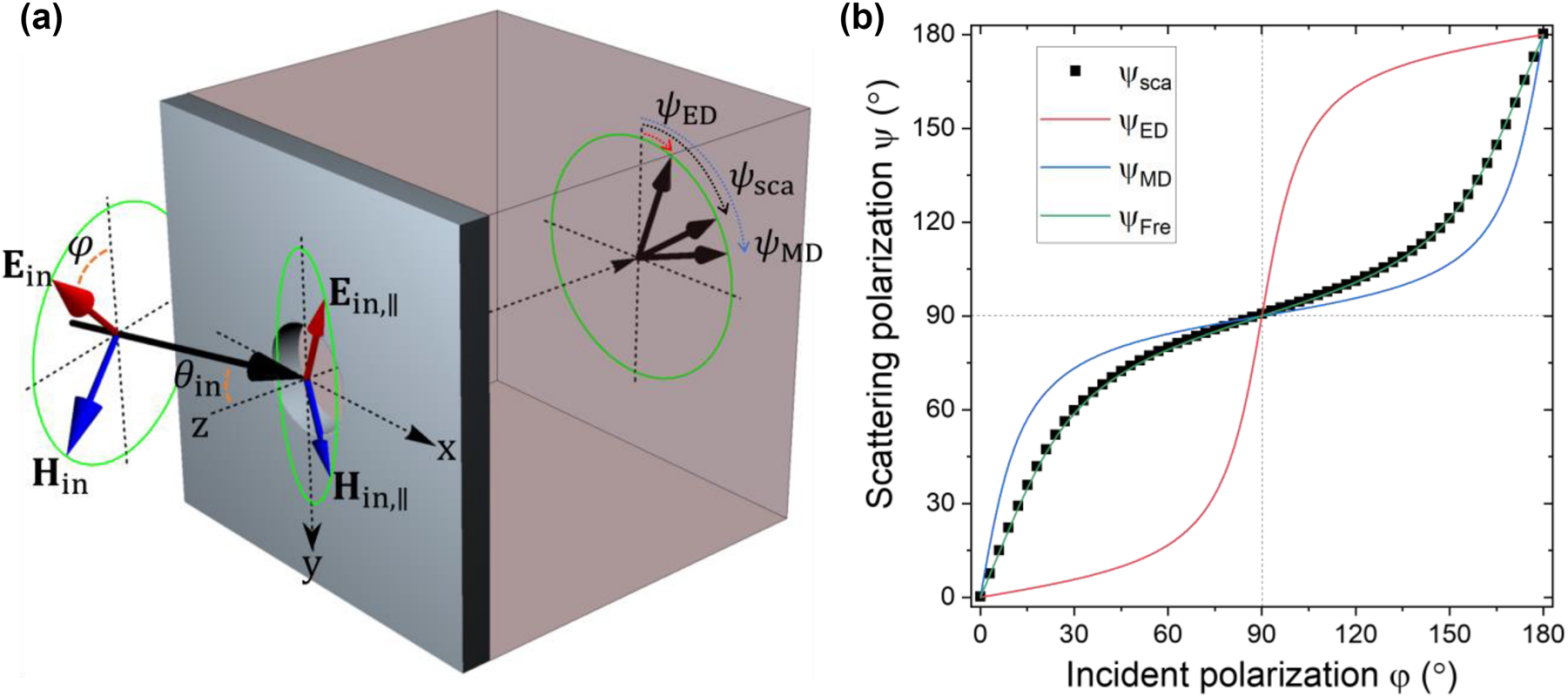
Magnetic dipole-like scattering at oblique incidence. (a) Schematic illustration of scattering with an incident angle of 80°. In this situation, angle between projections of incident electric and magnetic fields on the film plane is larger than 90°, which implies that in-plane electric and magnetic dipoles driven by incident electric and magnetic fields respectively will radiate different polarizations (*ψ*_ED_, *ψ*_MD_). Therefore, which of the two dipole polarizations is closer to the actual scattering polarization *ψ*_sca_ reveals the nature of the scattering. (b) Simulation results on scattering polarization for the oblique incidence. Black dots are the calculated scattering polarization. For comparison, *ψ*_ED_, *ψ*_MD_, and *ψ*_Fre_ are also plotted as red, blue, and green lines, respectively. *ψ*_Fre_ is the angle given by Fresnel equations for the air/Si interface.

The magnetic dipole response becomes even more evident when investigating the scattering polarization at oblique incidence. Investigation on scattering polarization at oblique incidence has been exploited in the previous studies [9–11] to distinguish an electric dipole and a magnetic dipole. Considering the normal detection scheme shown in Figure 2(a), a magnetic dipole induced by the in-plane magnetic component **H**_in,‖_ will have the scattering polarization *ψ*_MD_ perpendicular to **H**_in,‖_, while an electric dipole driven by the in-plane electric component **E**_in,‖_ will have the polarization *ψ*_ED_ of the same direction. For normal incidence, these two polarizations cannot be distinguished (*ψ*_MD_ = *ψ*_ED_) because **H**_in,‖_ and **E**_in,‖_ are perpendicular to each other. However, for oblique incidence with an incidence angle *θ*_in_ and polarization angle *φ*, the in-plane components are given by $H_{\text{in},\|}=\left(\cos\varphi \cos\theta_{\text{in}}\hat{x}+\text{sin}\;\varphi \hat{y}\right)H_{\text{in}}$ and $E_{\text{in},\|}=\left(\text{sin}\;\varphi \cos\theta_{\text{in}}\hat{x}-\cos\varphi \hat{y}\right)E_{\text{in}}$ , and accordingly, $\psi_{\text{MD}}={\text{tan}}^{-1}\left(\sin\varphi /\cos\varphi \cos\theta_{\text{in}}\right)$ and $\psi_{\text{ED}}={\text{tan}}^{-1}\left(\sin\varphi \cos\theta_{\text{in}}/\cos\varphi \right)$ which are distinguishable from each other.

We investigated scattering polarization *ψ*_sca_ at the normal scattering direction with an incident angle of 80°. Except the incident angle, rest of the simulation condition was the same as above. The simulated scattering polarization shown in Figure 2(b) was much closer to *ψ*_MD_ than *ψ*_ED_, indicating that the Si aperture virtually response to UV as a magnetic dipole. Moreover, we found that the scattering polarization can be predicted much more precisely by calculating Fresnel equations at the air/Si interface. A close relation between Fresnel equations and magnetic response of a metal aperture had been pointed out in previous studies [12, 13]. We decomposed the incident polarization into s- and p-polarization components, and the in-plane electric field for the respective polarizations was obtained using Fresnel equations at the interface as follows: $E_{y}=-\cos\varphi \left(1+r_{\text{s}}\left(\theta_{\text{in}}\right)\right)E_{\text{in}}$ and $E_{x}=\text{sin}\;\varphi \cos\theta_{\text{in}}\left(1-r_{\text{p}}\left(\theta_{\text{in}}\right)\right)E_{\text{in}}$ where *r*_s,p_ are amplitude reflection coefficients. From the polarization ellipse given by *E*_*x*_ and *E*_*y*_ at the infinite air/Si interface, we deduced angle of the major axis *ψ*_Fre_, which is plotted as a function of incident polarization in Figure 2(b) showing excellent agreement with the simulated hole scattering polarization *ψ*_sca_. It can be understood from the fact that, for a highly absorbing plasmonic film thicker than the penetration depth, light barely reaches the exit side film/substrate interface, so the reflection at the film/substrate interface cannot affect the overall polarization inside the film. Accordingly, scattering from a small hole in a such plasmonic film has polarization simply duplicating the in-plane polarization solely determined by the incident side interface (with a possible phase difference). Because the penetration depth given by imaginary part of the Si refractive index *λ*/2π*κ* is about 16 nm, a Si thickness of only few tens of nanometers is enough to satisfy the above condition at the UV wavelength. The Si thickness of 80 nm assumed in Figure 2(b) is already much thicker than the penetration depth and we can expect that a small hole on a Si film thicker than 80 nm will also show scattering polarization close to *ψ*_Fre_ because the same physics will apply. On the other hand, scattering polarization from a small hole on a film thinner than the penetration depth can significantly deviate from *ψ*_Fre_ due to the reflection at the film/substrate interface (see Supplementary Materials S2). We also note that a simple algebra shows that *ψ*_Fre_ is recovered to *ψ*_MD_ when the refractive index of the film is infinite, agreeing with Bethe’s theory. That is, the similarity between *ψ*_Fre_ and *ψ*_MD_ for the Si hole is a direct consequence of the high complex refractive index ( ${\tilde{n}}_{310\text{nm}}=2.87+3.06i$ ) at the UV wavelength.

**Figure 3:**
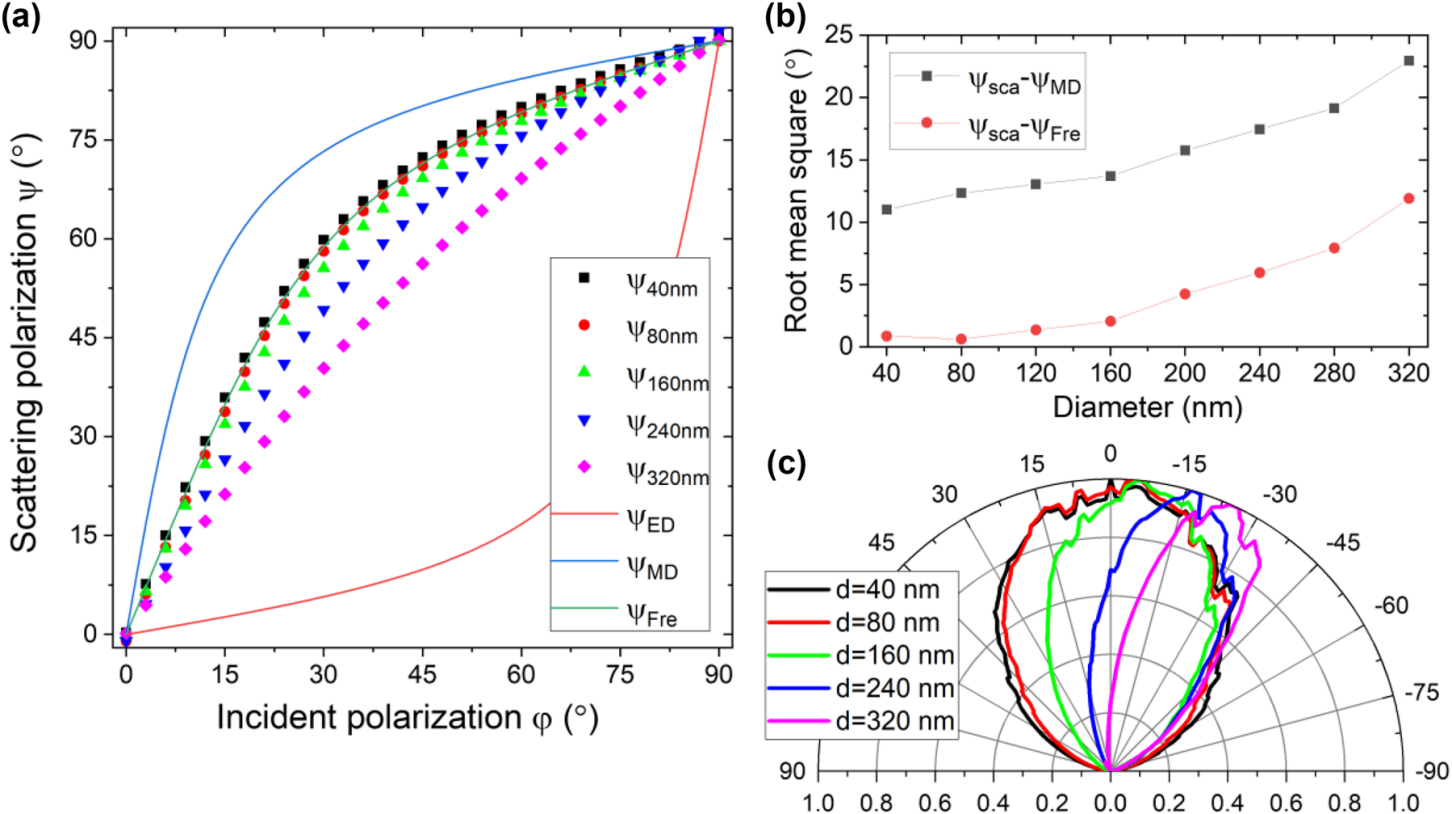
Simulated diameter dependence of UV scattering by Si apertures. (a) Scattering polarization obtained for various diameters from 40 to 320 nm. (b) Root mean square of differences, *ψ*_sca_ − *ψ*_MD_ and *ψ*_sca_ − *ψ*_Fre_, calculated from the values given in (a). (c) Scattering patterns in the xz plane for the TE incidence polarization (*φ* = 0).

We investigated diameter dependence of the magnetic dipole-like scattering to check the meaning of ‘small holes’ for UV plasmonics of Si. Figure 3(a) shows simulated scattering polarization curves for diameters up to 320 nm which is slightly over the wavelength *λ* of 310 nm. The curves for diameters of 40 and 80 nm are very close to the curve *ψ*_Fre_ given by Fresnel equations. However, polarization curves start to deviate from *ψ*_Fre_ when diameter increases further and scattering polarization by a 320 nm-hole almost loses the feature of a magnetic dipole, being *ψ*_320nm_ ∼ *φ*. We quantified this deviation by calculating root mean squares of *ψ*_sca_ − *ψ*_Fre_ and *ψ*_sca_ − *ψ*_MD_. As shown in Figure 3(b), *ψ*_sca_ − *ψ*_Fre_ is negligible when diameter is below 80 nm (∼*λ*/4), but when diameter is over 160 nm (∼*λ*/2), the deviation become increasingly noticeable. Therefore, in terms of scattering polarization, holes with a diameter less than *λ*/4 can be regarded as ‘small holes’ behaving similar to a magnetic dipole.

We checked the criterion, $\left(\text{diameter}\right)<\lambda /4$ , again in terms of scattering pattern. Figure 3(c) shows simulated scattering patterns for the various diameters. For diameters of 40 and 80 nm, the scattering patterns were almost same with the cosine squared, confirming the criterion. When diameter increases over 160 nm, the scattering pattern deviates from the cosine squared to a more directional shape implying contribution of higher order multipoles. Because the cutoff wavelength of the fundamental TE_11_ mode in a PEC circular waveguide is given by $\lambda_{\text{cutoff}}=\left(\text{diameter}\right)\pi /1.841$ , the significant higher order contribution already at ∼*λ*/2 indicates that Si play as a real metal at the UV wavelength, making the effective hole diameter larger than the nominal due to the field penetration [25].

## 3 Experimental measurements

**Figure 4:**
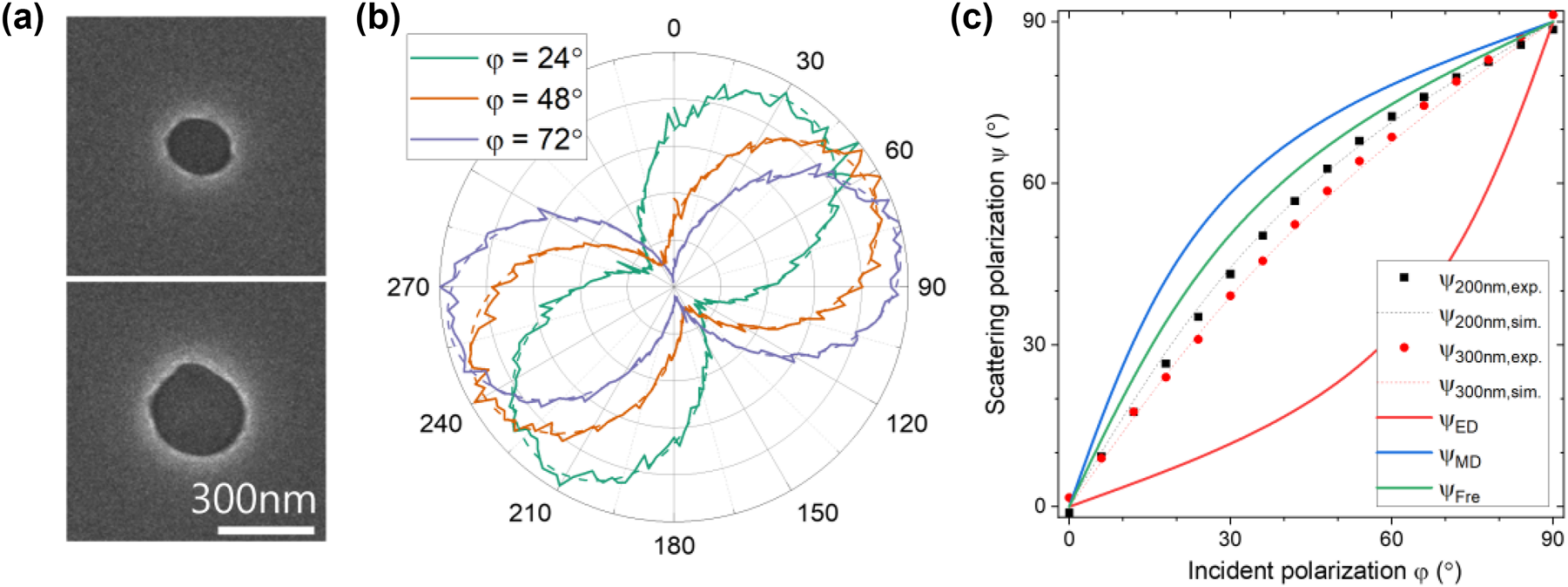
Experimental confirmation of the theoretical analysis. (a) Scanning electron microscope images of apertures on a Si film of 75 nm thickness. Diameter of the upper is 200 nm and the lower is 300 nm. (b) Typical polar plots of polarization analyzed scattering intensity from the 200 nm Si hole for various incident polarizations. The incident angle is 69° and the wavelength is 365 nm. Dashed lines are fitting curves. (c) Scattering polarization versus incident polarization. Black and red dots for diameters of 200 and 300 nm, respectively. Simulation results (dotted lines) and *ψ*_ED_, *ψ*_MD_, and *ψ*_Fre_ (soled lines) are also plotted for comparison.

We measured scattering polarization from Si apertures with oblique UV incidence (*θ*_in_ = 69°) to confirm the theoretical analysis discussed above. Samples fabricated on a c-cut sapphire substrate are shown in Figure 4(a). Fabrication and experiment details are given in the methods section. Because the *c*-axis in a c-cut sapphire is normal to the surface, the birefringence effect was removed in the detection direction. For various incident polarizations in the range of 0° to 90°, we analyzed scattering polarizations, some of which are presented in Figure 4(b). Since we used small numerical aperture of 0.1 for scattering collection, the measurements can be regarded to have been performed only for scattering in the normal direction, being consistent with the simulations. The measured intensities showed an excellent sinusoidal modulation depending on angle of the analyzing polarizer, enabling extraction of well-defined scattering polarization by fitting with a sinusoidal function (dashed lines, Figure 4(b)).

Scattering polarizations obtained by fitting are plotted in Figure 4(c) as a function of incident polarization. Scattering polarizations from the 200 nm-hole and the 300 nm-hole both show some magnetic dipole-like responses, being closer to *ψ*_MD_ than *ψ*_ED_. We note that, the difference between *ψ*_MD_ and *ψ*_ED_ in Figure 4(c) is smaller than the one given in the above section because of the smaller incident angle. Scattering polarization curve expected from Fresnel equations, *ψ*_Fre_, is quite close to *ψ*_MD_ due to the high complex refractive index ( ${\tilde{n}}_{365\text{nm}}=3.90+2.66i$ ) implying that scattering will be more magnetic dipole-like if experiment is performed with smaller holes. Deviation of experimental scattering polarization curves from *ψ*_Fre_ can be attributed to the fact that the diameters of 200 and 300 nm are already over half the wavelength. Scattering polarization from the 300 nm-hole show larger deviation than the one from the 200 nm-hole as we discussed in the above section. We performed simulations with the experimental conditions and the results (dotted lines, Figure 4(c)) were in good agreement, confirming the validity of our simulation method. The difference between the experiments and simulations can be partially attributed to the slight ellipticity and irregularity of the fabricated holes (see Supplementary Materials S3).

## 4 Conclusions

Magnetic dipole-like response of Si apertures in the UV range was demonstrated in terms of scattering pattern and polarization. If the hole diameter is small, the scattering pattern is given by cosine squared like a dipole radiation, and the scattering polarization determined by Fresnel equations at the incident side interface is similar to the orientation perpendicular to the in-plane component of the incident magnetic field. In other words, refractive index of Si at the UV wavelength is sufficiently large to reproduce magnetic dipole responses of a metallic hole at longer wavelengths predicted by Bethe’s theory. Our work shows that Si is a promising plasmonic material in the UV range and scattering polarization from a small hole can be predicted using Fresnel equations valid for any plasmonic materials, paving a way for UV magnetism and UV magnetic field mapping.

## 5 Methods

### 5.1 Simulation

We used a commercial simulation software, COMSOL Multiphysics 6.1 with the wave optics module. The simulation space was a sphere with a radius of 2.2*λ* and a perfectly matched layer covered the sphere with a thickness of 1*λ*. The sapphire substrate occupied a half sphere, and the other half was occupied by the Si film and air. The center of hole bottom face was set to the origin. To implement scattering by a single hole, scattered field formulation was applied with the background field calculated for the air/Si film/sapphire system except a hole. Because we adopted scattered field formulation, we regarded the relative fields, which are defined as difference between the total field and the background field in COMSOL, as the scattered fields. Scattering pattern was obtained at the distance of 2*λ* from the origin in the xz plane. That is, we obtained time-averaged power flow of the relative fields in the normal direction of the half circle of 2*λ* radius. Scattering polarization was also calculated at the 2*λ* distance by taking the major axis of the polarization ellipse. Maximum mesh size in the Si film was set as the penetration depth. We used refractive index of amorphous Si ( $\tilde{n}=2.87+3.06i$ at *λ* = 310 nm and $\tilde{n}=3.90+2.66i$ at *λ* = 365 nm) [26] and refractive index of the sapphire substrate was set as 1.79.

### 5.2 Experiment

For sample preparation, we deposited 75 nm thick Si and 20 nm thick Cu films sequentially on a c-cut sapphire substrate using electron beam evaporation. The holes were fabricated by focused ion beam (FIB) milling where the Cu film prevented charging effect. After finishing the FIB milling, the Cu film was removed by CR-7 etchant. For optical experiment, UV from a 365 nm light emitting diode (Thorlabs, M365L2) was collimated by an aspheric condenser lens. The UV beam passed a polarizer and a half-wave plate defining incident polarization *φ* and was weakly focused onto the sample from the air side with the incident angle of 69°. Scattering in the normal direction was collected by an objective lens with a numerical aperture of 0.1. The image on the conjugate focal plane was cropped by an iris diaphragm to collect scattering only from the hole region which was identified by a CCD camera. The cropped scattering was re-focused on a single photon counting module (Perkinelmer, SPCM-AQR-14), whose electrical signal was read by a photon counter (SRS, SR400). Background noise from a region without holes was also measured to calculate net scattering signal from the hole. Scattering polarization was analyzed by rotating a motorized polarizer before the detector with a 2° step. The polarization analyzed data were fitted to $I\left(\psi \right)=a+b\cos2\left(\psi -\psi_{\text{sca}}\right)$ to extract the scattering polarization *ψ*_sca_.

## Supplementary Material

Supplementary Material Details

## References

[1] Genet C., Ebbesen T. W. (2007). Light in tiny holes. *Nature*.

[2] Zhang Y., Han M., Huang C.-P. (2021). Plasmon coupling in circular-hole dimers: from separation- to touching-coupling regimes. *J. Appl. Phys.*.

[3] Dao T. D., Ishii S., Yokoyama T. (2016). Hole array perfect absorbers for spectrally selective midwavelength infrared pyroelectric detectors. *ACS Photonics*.

[4] Choy J. T., Hausmann B. J. M., Babinec T. M. (2011). Enhanced single-photon emission from a diamond–silver aperture. *Nat. Photonics*.

[5] Rigneault H., Capoulade J., Dintinger J. (2005). Enhancement of single-molecule fluorescence detection in subwavelength apertures. *Phys. Rev. Lett.*.

[6] Langguth L., Punj D., Wenger J., Koenderink A. F. (2013). Plasmonic band structure controls single-molecule fluorescence. *ACS Nano*.

[7] Lee S. H., Bantz K. C., Lindquist N. C., Oh S.-H., Haynes C. L. (2009). Self-assembled plasmonic nanohole arrays. *Langmuir*.

[8] Bethe H. A. (1944). Theory of diffraction by small holes. *Phys. Rev.*.

[9] Kihm H. W., Koo S. M., Kim Q. H. (2011). Bethe-hole polarization analyser for the magnetic vector of light. *Nat. Commun.*.

[10] Kihm H. W., Kim J., Koo S. (2013). Optical magnetic field mapping using a subwavelength aperture. *Opt. Express*.

[11] Singh D. K., Ahn J. S., Koo S. (2015). Selective electric and magnetic sensitivity of aperture probes. *Opt. Express*.

[12] Lee D., Kim D.-S. (2016). Light scattering of rectangular slot antennas: parallel magnetic vector vs perpendicular electric vector. *Sci. Rep.*.

[13] Yang H., Kim D.-S., Kim R. H. J.-Y. (2018). Magnetic nature of light transmission through a 5-nm gap. *Sci. Rep.*.

[14] Barulin A., Claude J.-B., Patra S., Bonod N., Wenger J. (2019). Deep ultraviolet plasmonic enhancement of single protein autofluorescence in zero-mode waveguides. *Nano Lett*..

[15] Watson A. M., Zhang X., Alcaraz de la Osa R. (2015). Rhodium nanoparticles for ultraviolet plasmonics. *Nano Lett*..

[16] Ghori M. Z., Veziroglu S., Hinz A. (2018). Role of UV plasmonics in the photocatalytic performance of TiO_2_ decorated with aluminum nanoparticles. *ACS Appl. Nano Mater.*.

[17] Deng Y., Wang X., Gong Z. (2018). All-silicon broadband ultraviolet metasurfaces. *Adv. Mater.*.

[18] Xu T., Agrawal A., Abashin M., Chau K. J., Lezec H. J. (2013). All-angle negative refraction and active flat lensing of ultraviolet light. *Nature*.

[19] Shen K.-C., Ku C.-T., Hsieh C., Kuo H.-C., Cheng Y.-J., Tsai D. P. (2018). Deep-ultraviolet hyperbolic metacavity laser. *Adv. Mater.*.

[20] Chou Y.-H., Hong K.-B., Chang C.-T. (2018). Ultracompact pseudowedge plasmonic lasers and laser arrays. *Nano Lett*..

[21] Barulin A., Claude J.-B., Patra S., Moreau A., Lumeau J., Wenger J. (2019). Preventing aluminum photocorrosion for ultraviolet plasmonics. *J. Phys. Chem. Lett.*.

[22] Knight M. W., Coenen T., Yang Y. (2015). Gallium plasmonics: deep subwavelength spectroscopic imaging of single and interacting gallium nanoparticles. *ACS Nano*.

[23] Dong Z., Wang T., Chi X. (2019). Ultraviolet interband plasmonics with Si nanostructures. *Nano Lett*..

[24] Shekhar P., Pendharker S., Sahasrabudhe H. (2018). Extreme ultraviolet plasmonics and Cherenkov radiation in silicon. *Optica*.

[25] Gordon R., Brolo A. G. (2005). Increased cut-off wavelength for a subwavelength hole in a real metal. *Opt. Express*.

[26] Pierce D. T., Spicer W. E. (1972). Electronic structure of amorphous Si from photoemission and optical studies. *Phys. Rev. B*.

