# Supplementary material for: Ultraviolet light scattering by a silicon Bethe hole
Source: Nanophotonics. 2023 Nov 6;13(7):1091–7. doi: 10.1515/nanoph-2023-0557 (PMC11501262; doi:10.1515/nanoph-2023-0557)
Supplement: Supplementary file 1 — Supplementary Material Details [file j_nanoph-2023-0557_suppl_001.docx]

Supplementary Materials

**Ultraviolet light scattering by a silicon Bethe hole**

Dukhyung Lee, Youjin Lee and Dai-Sik Kim*

*Corresponding author: **Dai-Sik Kim**, Ulsan National Institute of Science and Technology, Ulsan 44919, Republic of Korea; daisikkim@unist.ac.kr

**Dukhyung Lee**: Ulsan National Institute of Science and Technology, Ulsan 44919, Republic of Korea; hyung0624@unist.ac.kr

**Youjin Lee**: Seoul National University, Seoul 08826, Republic of Korea; lyj3199@snu.ac.kr

**Table of Contents**

S1. Scattered magnetic field components at the normal incidence.

S2. Scattering polarization for various Si thicknesses.

S3. Scattering polarization from elliptical Si holes.

**S1. Scattered magnetic field components at the normal incidence.**


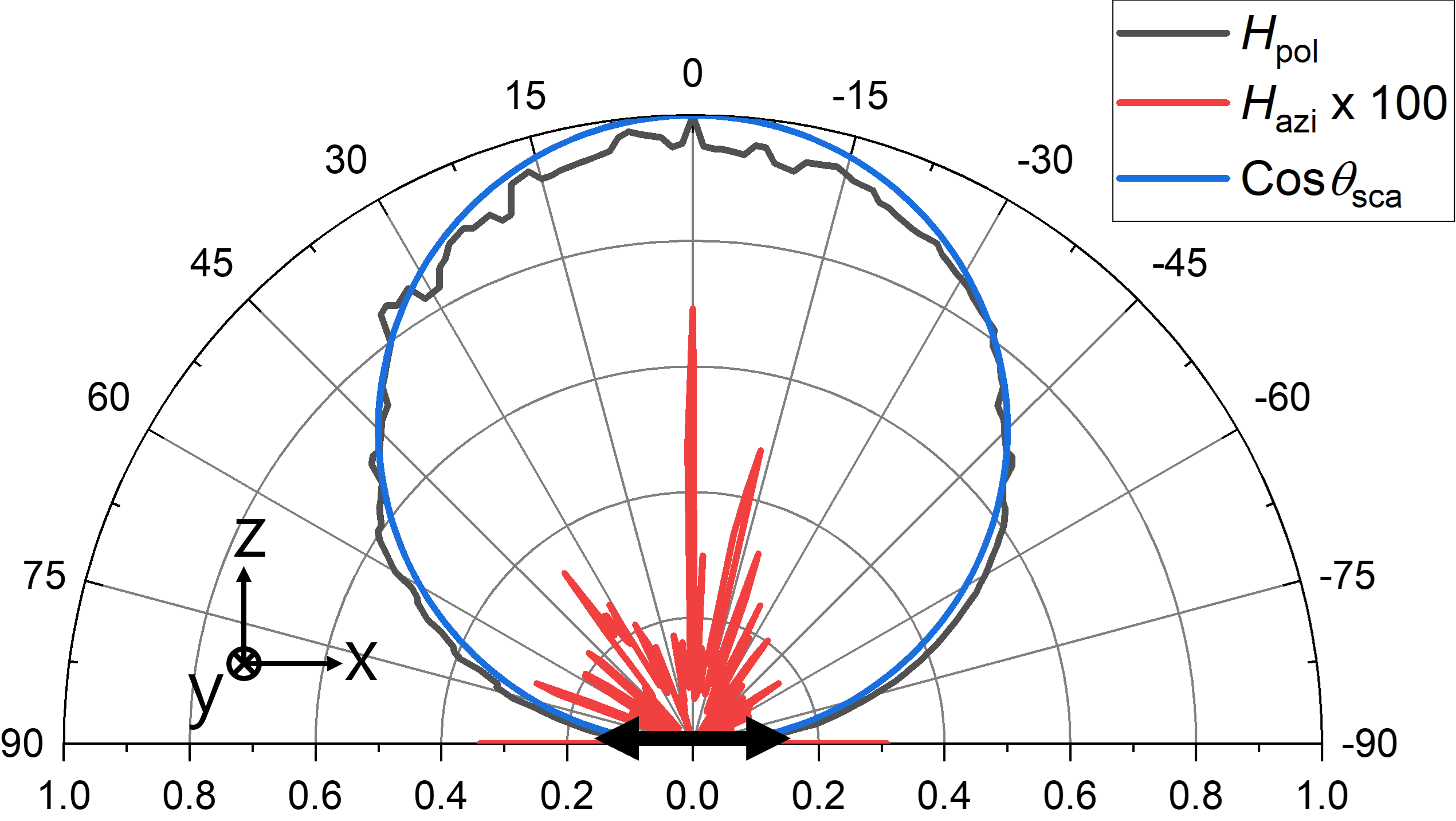


Fig S1. Polar plot of the simulated polar and azimuthal components of the scattered magnetic field ($\boldsymbol{H}_{\mathbf{pol}}$, $\boldsymbol{H}_{\mathbf{azi}}$) obtained at the $\boldsymbol{2}\boldsymbol{\lambda}$ distance in the xz plane and normalized by the maximum $\boldsymbol{H}_{\mathbf{pol}}$. Cosine function plotted together represents polar/azimuthal magnetic field of the radiation from a magnetic/electric dipole directed along the x-axis, which is denoted as the black arrow.

We calculated the scattered polar and azimuthal (y-direction on the xz plane) magnetic fields at the normal incidence discussed in Figure 1 in the main text. Although the scattering intensity pattern given in Figure 1(c) can be generated by both electric and magnetic dipoles directed along the x-axis, investigation on polarization, or field components, tells the nature of the dipole. For the y-polarized normal incidence, the scattered polar magnetic field $H_{\mathrm{pol}}$ agrees well with $\cos\theta_{\mathrm{sca}}$ which is expected from a magnetic dipole directed along the x-axis. Meanwhile, the scattered azimuthal magnetic field $H_{\mathrm{azi}}$ shows negligible amplitude and an irregular pattern, implying that it is essentially a simulation artifact, not an electric dipole radiation which also should be $\cos\theta_{\mathrm{sca}}$.

**S2. Scattering polarization for various Si thicknesses.**


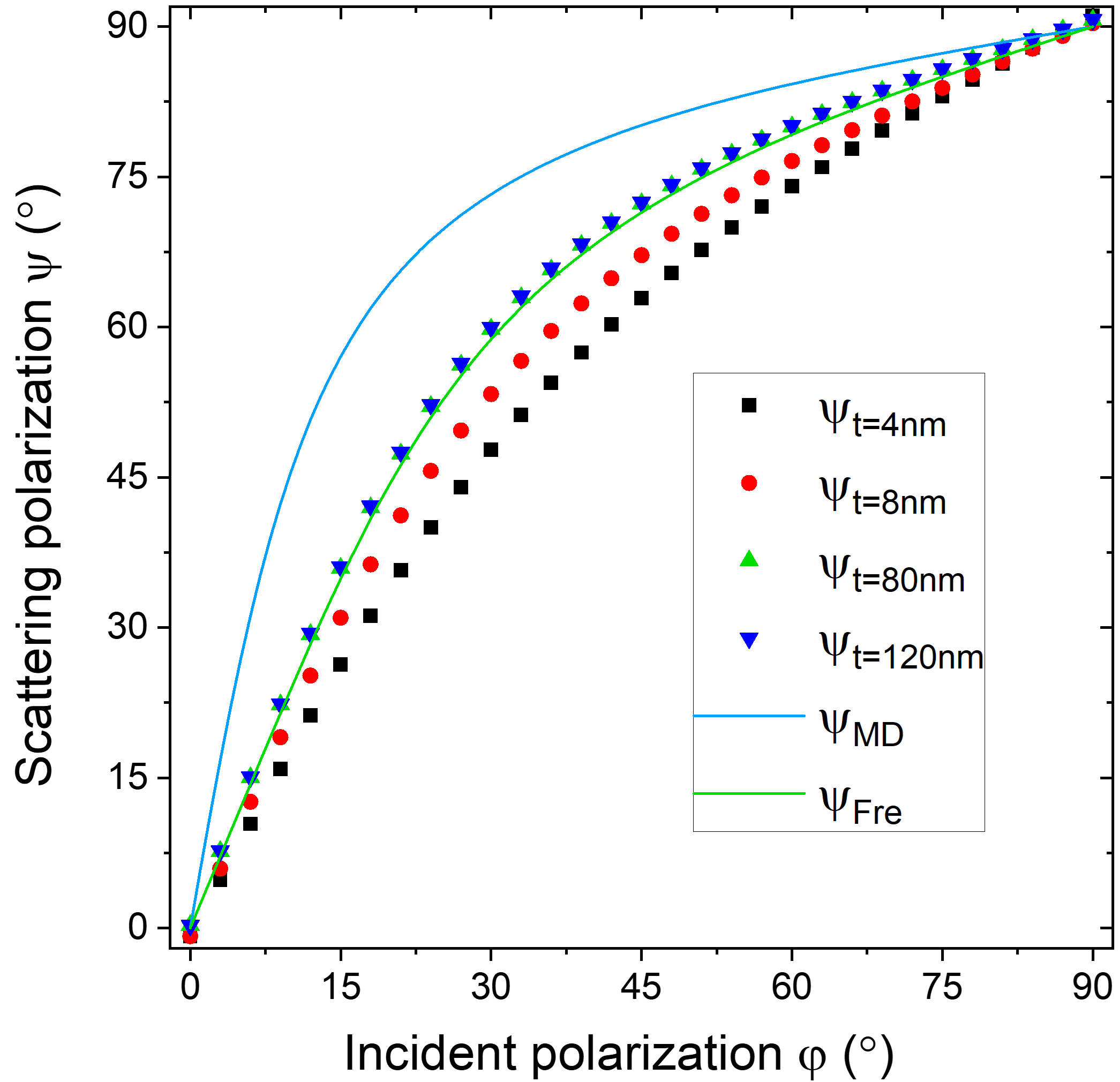


Fig. S2. Scattering polarization versus incident polarization for Si thicknesses of 4, 8, 80, and 120 nm. Rest of the simulation condition was the same as in Fig. 2(b) in the main text. That is, $\boldsymbol{\lambda}$ = 310 nm, $\boldsymbol{\theta}_{\mathbf{in}}$ = 80°, and the diameter is 40 nm.

For thicknesses of 80 and 120 nm which are much thicker than the penetration depth of 16 nm, the scattering polarizations are almost same with the polarization $\psi_{\mathrm{Fre}}$ given by Fresnel equations at the air/Si interface, as discussed in the main text. That is, $\psi_{\mathrm{Fre}}$ determines the limit of magnetic dipole-like response of a small hole from the point of view of scattering polarization. When the thickness is smaller than the penetration depth, reflection at the Si/substrate interface comes into play, resulting in deviation of scattering polarization from $\psi_{\mathrm{Fre}}$, as shown by the black (4-nm thickness) and red (8-nm thickness) dots in Figure S2.

**S3. Scattering polarization from elliptical Si holes.**


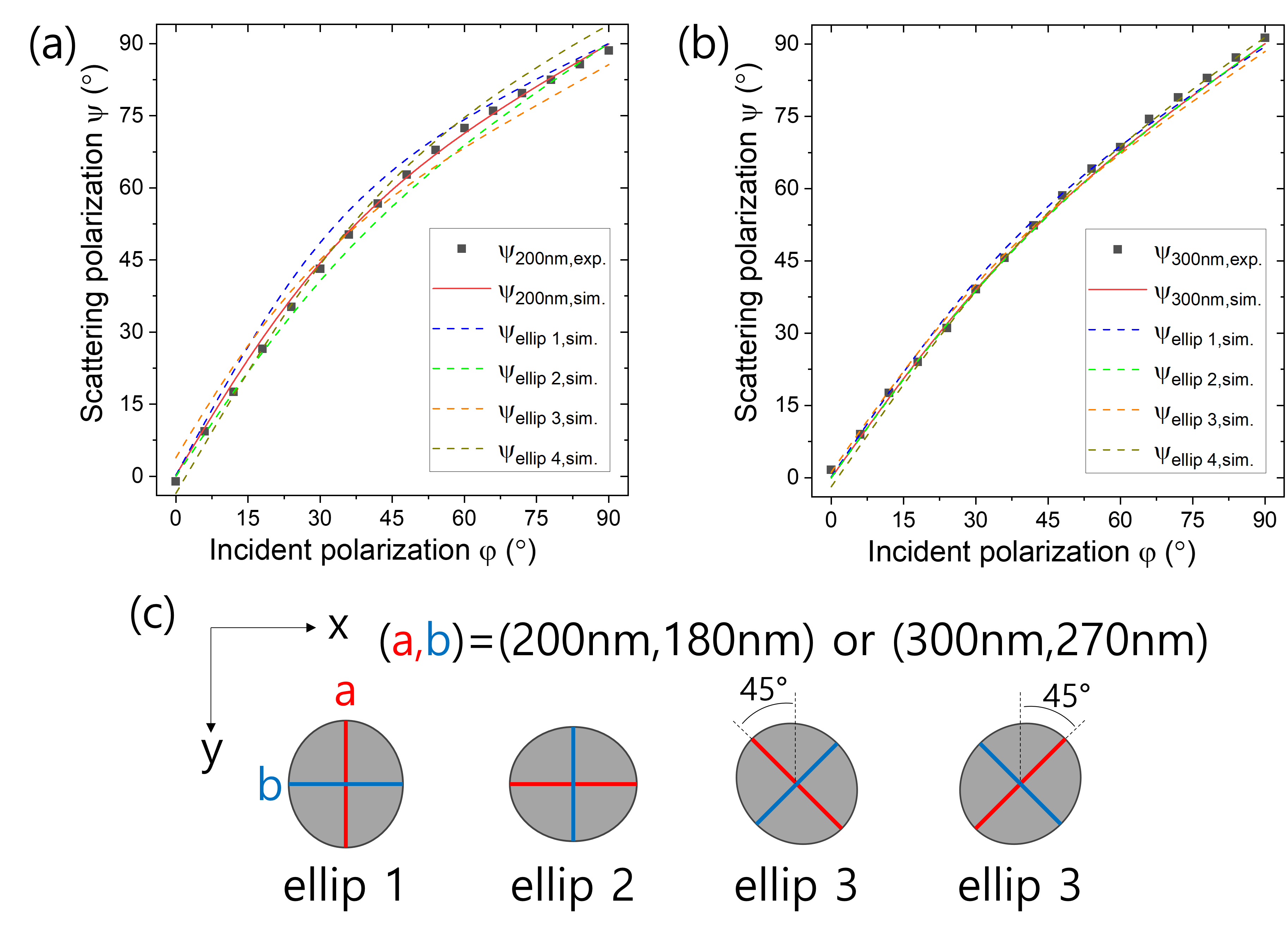


Fig. S3. (a,b) Comparison between the experimental scattering polarizations and simulated scattering polarizations from circular and elliptical holes. Nominal diameters in the experiment were (a) 200 nm and (b) 300 nm. (c) The 4 elliptical cases considered in (a,b).

We calculated scattering polarizations from elliptical holes whose dimensions are similar to the ones identified in the SEM images in Figure 4(a) in the main text. Figure S3 shows that the elliptical shapes result in a small deviation from the circular case, which might account for a fraction of the experimental error.
